# Single Nucleotide Polymorphism Clustering in Systemic Autoimmune Diseases

**DOI:** 10.1371/journal.pone.0160270

**Published:** 2016-08-04

**Authors:** Thomas Charlon, Manuel Martínez-Bueno, Lara Bossini-Castillo, F. David Carmona, Alessandro Di Cara, Jérôme Wojcik, Sviatoslav Voloshynovskiy, Javier Martín, Marta E. Alarcón-Riquelme

**Affiliations:** 1 Quartz Bio, Plan-les-Ouates, Geneva, Switzerland; 2 Stochastic Information Processing, University of Geneva, Geneva, Geneva, Switzerland; 3 Center for Genomics and Oncological Research: Pfizer/University of Granada/Andalusian Government, Granada, Granada, Spain; 4 Institute of Parasitology and Biomedicine López Neyra, Spanish National Research Council, Armilla, Granada, Spain; Hospital Israelita Albert Einstein, BRAZIL

## Abstract

Systemic Autoimmune Diseases, a group of chronic inflammatory conditions, have variable symptoms and difficult diagnosis. In order to reclassify them based on genetic markers rather than clinical criteria, we performed clustering of Single Nucleotide Polymorphisms. However naive approaches tend to group patients primarily by their geographic origin. To reduce this “ancestry signal”, we developed SNPClust, a method to select large sources of ancestry-independent genetic variations from all variations detected by Principal Component Analysis. Applied to a Systemic Lupus Erythematosus case control dataset, SNPClust successfully reduced the ancestry signal. Results were compared with association studies between the cases and controls without or with reference population stratification correction methods. SNPClust amplified the disease discriminating signal and the ratio of significant associations outside the *HLA* locus was greater compared to population stratification correction methods. SNPClust will enable the use of ancestry-independent genetic information in the reclassification of Systemic Autoimmune Diseases. SNPClust is available as an R package and demonstrated on the public Human Genome Diversity Project dataset at https://github.com/ThomasChln/snpclust.

## Introduction

The PRECISESADS project aims at reclassifying Systemic Autoimmune Diseases (SADs), a group of chronic inflammatory conditions characterized by the presence of unspecific autoantibodies in the serum and serious clinical consequences, based on genetic and molecular biomarkers rather than clinical criteria. SADs affect 1% of the global population [1] and have limited treatment options and difficult diagnosis. The diseases studied in PRECISESADS are Systemic Lupus Erythematosus (SLE), Systemic Sclerosis, Rheumatoid Arthritis, Sjögren’s Syndrome, Primary Antiphospholipid Antibody Syndrome, and undifferentiated cases.

Several technological platforms are used to generate biomarker data from patient samples, to obtain as much information as possible about the genetic and molecular mechanisms involved. The technologies include Single Nucleotide Polymorphisms (SNPs) microarrays, which measures hundreds of thousands of common genetic variations in a population, gene expression and protein microarrays, mass spectrometry for metabolic profiling, or flow cytometry, as examples. The methodological approach is to first analyze data from each technological platform individually and then to merge relevant features from each platform to build the final classifier. Here we present the results of the preparatory work for SNP clustering analysis performed on a test dataset.

Familial and twin studies have estimated a 50% genetic component in SADs [2, 3] and Genome-Wide Association Studies (GWAS) have found several loci associated with SADs [4]. However, genome-wide clustering of SNPs is known to primarily group patients by ancestry prior to disease relevant features [5, 6]. In order to emphasize the disease relevant signal, we developed SNPClust, a clustering method to overcome this “ancestry bias” by selecting and summarizing SNPs contributing strongly to localized sources of genetic variation as detected by Principal Component Analysis (PCA) [7].

SNPClust first applied PCA to project patients on the largest sources of variance by linear combinations of SNPs. Then for each principal component, the SNPs that had significantly high contributions were selected. Many correlated SNPs were selected from specific loci due to linkage disequilibrium between SNPs, which form haplotypes, and therefore still produced an ancestry signal. To address this, for SNPs selected from the same principal component, SNPClust summarized physically close SNPs in linkage disequilibrium by one variable inferring a haplotype, and reduced the ancestry signal while conserving the other underlying genetic signals.

The test dataset contained SNP microarray data from 4,212 European SLE patients and 1,221 European controls. After quality control, Minor Allele Frequency (MAF) filtering, and tag SNP selection [8], PCA was performed on 379,190 SNPs from 5,433 patients. For each of the 100 first principal components, strong contributing SNPs were selected and SNP-dense regions were summarized by haplotypes. A total of 261 SNPs were selected and 331 haplotypes inferred.

On the SNPClust selected dataset, the clustering signal due to ancestry was significantly reduced. The performance of SNPClust was compared to GWAS standard approaches and reference population substructure correction methods [9, 10]. SNPClust was shown to enrich the selection of ancestry-independent sources of genetic variation associated with the phenotype, and hence propose more robust candidate biomarkers.

## Results

### Ancestry clusters

**Fig 1:**
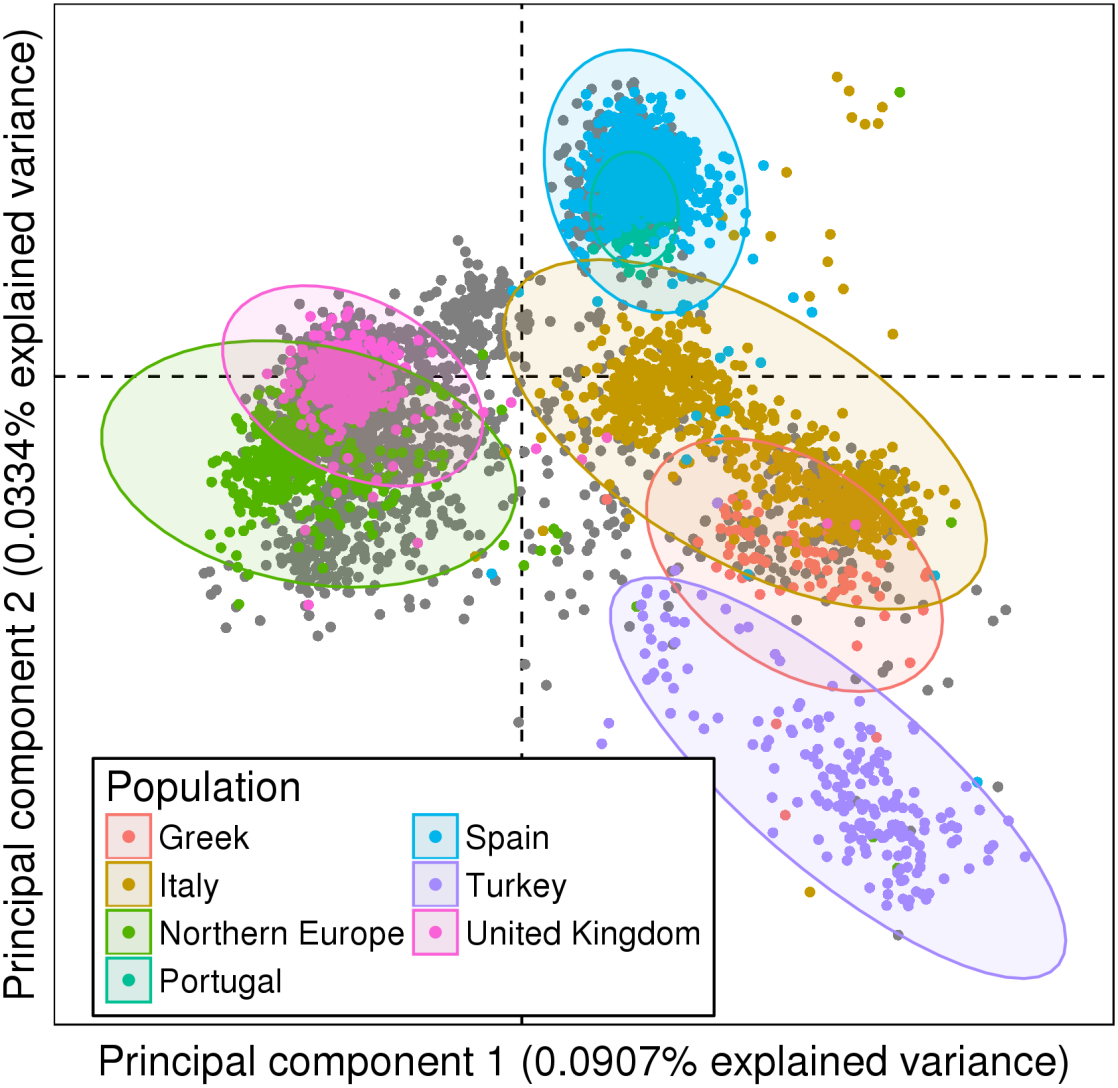
Initial grouping of genetic data. Two first principal components of the PCA on 379,190 SNPs from 5,433 European SLE patients and controls with 95% confidence ellipses. Northern and Southern Europeans were discriminated in the first principal component. Eastern and Western Europeans were discriminated in the second. 2,733 individuals (50%) did not have geographic information and were colored in gray.

As expected, PCA applied on the input dataset (without any prior feature selection or data transformation) grouped patients by the country of origin of the samples, discriminating Northern from Southern Europeans in the first principal component and Eastern from Western Europeans in the second (Fig 1).

This ancestry signal can also be seen in most of the 10 first principal components. The most important non-ancestry-based source of genetic variation in the PCA appeared on principal component 3 (see below), thus confirming that the ancestry signal is much stronger than clinically relevant signals in clustering approaches.

### Selection of strong contributors

**Fig 2:**
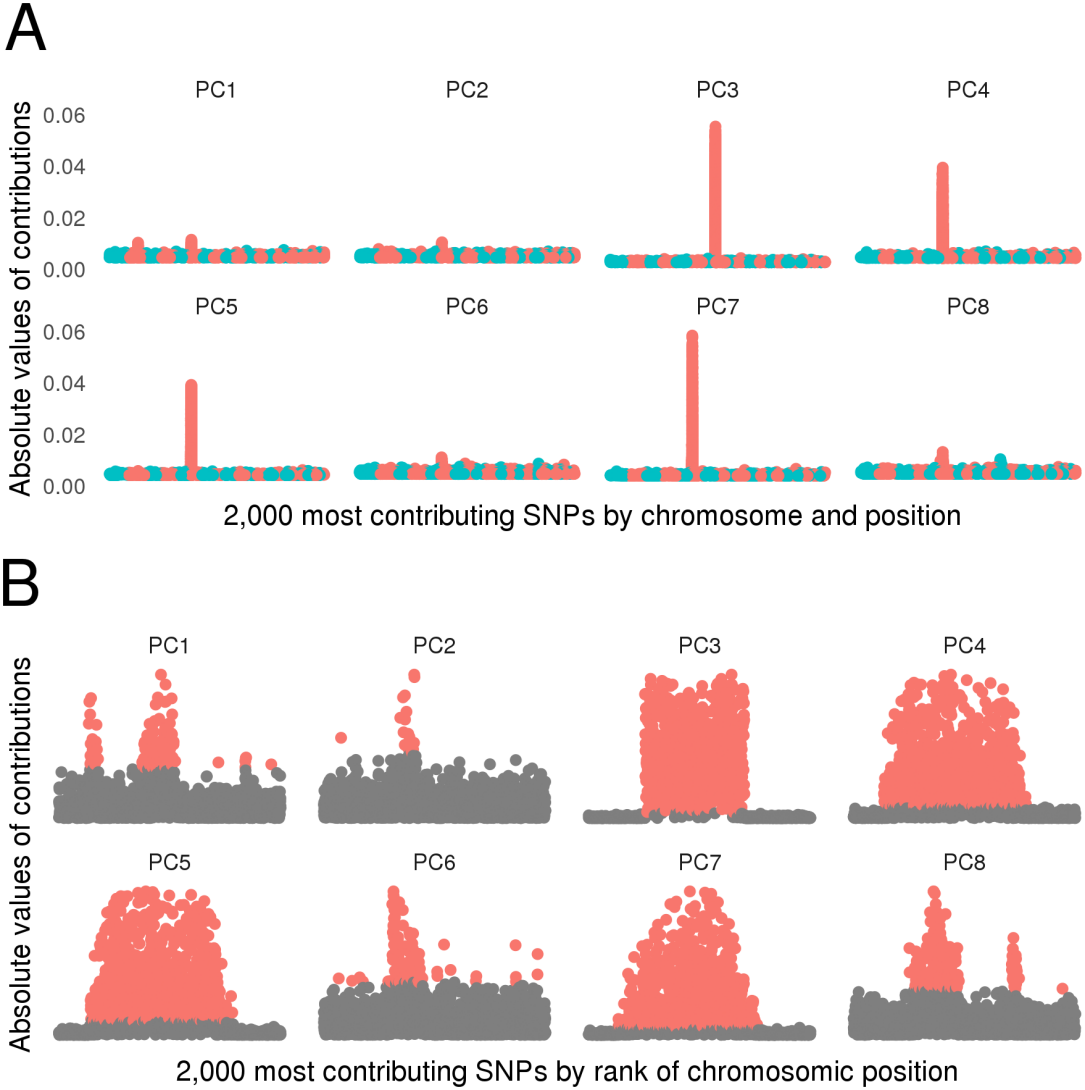
Selection of strong SNP contributors. **(a)** The 2,000 most contributing SNPs to each of the first 8 principal components are displayed by chromosomal position and colored by chromosomes. Principal components were driven by large localized SNP groups and the chromosome 6 locus *HLA* was the strongest and largest contributor in all of the first 8 principal components, except principal component 5. **(b)** Selection of SNPs by the Gaussian mixtures based method. Selected SNPs are colored in red. SNPs are displayed on the x-axis by rank of chromosomic position, *i.e.* SNPs are regularly spaced and ordered by chromosome and position.

The analysis of the contributions of SNP markers to the PCA principal component axes revealed that the first twenty principal components were driven by large localized SNP groups. The chromosome 6 *HLA* locus was the strongest and largest contributor in all of the first 8 principal components except principal component 3. Principal component 3 was driven by the chromosome 8 locus *8p23*, one of the largest inversion polymorphism encompassing 4,500,000 base pairs and including the SLE associated gene *BLK* [11] (Fig 2). As *8p23* was a main contributor in some of the first principal components, we confirmed that ancestry-independent signals can be extracted by SNPClust.

The 100 first principal components, on which the Gaussian mixture models based selection was applied, explained 3.5% of the total variance. Large localized SNP groups were selected along with other strong contributors. In total, 10,422 SNPs were selected, including 4,090 SNPs from the first 8 principal components (Fig 2).

### Haplotype summarization

**Fig 3:**
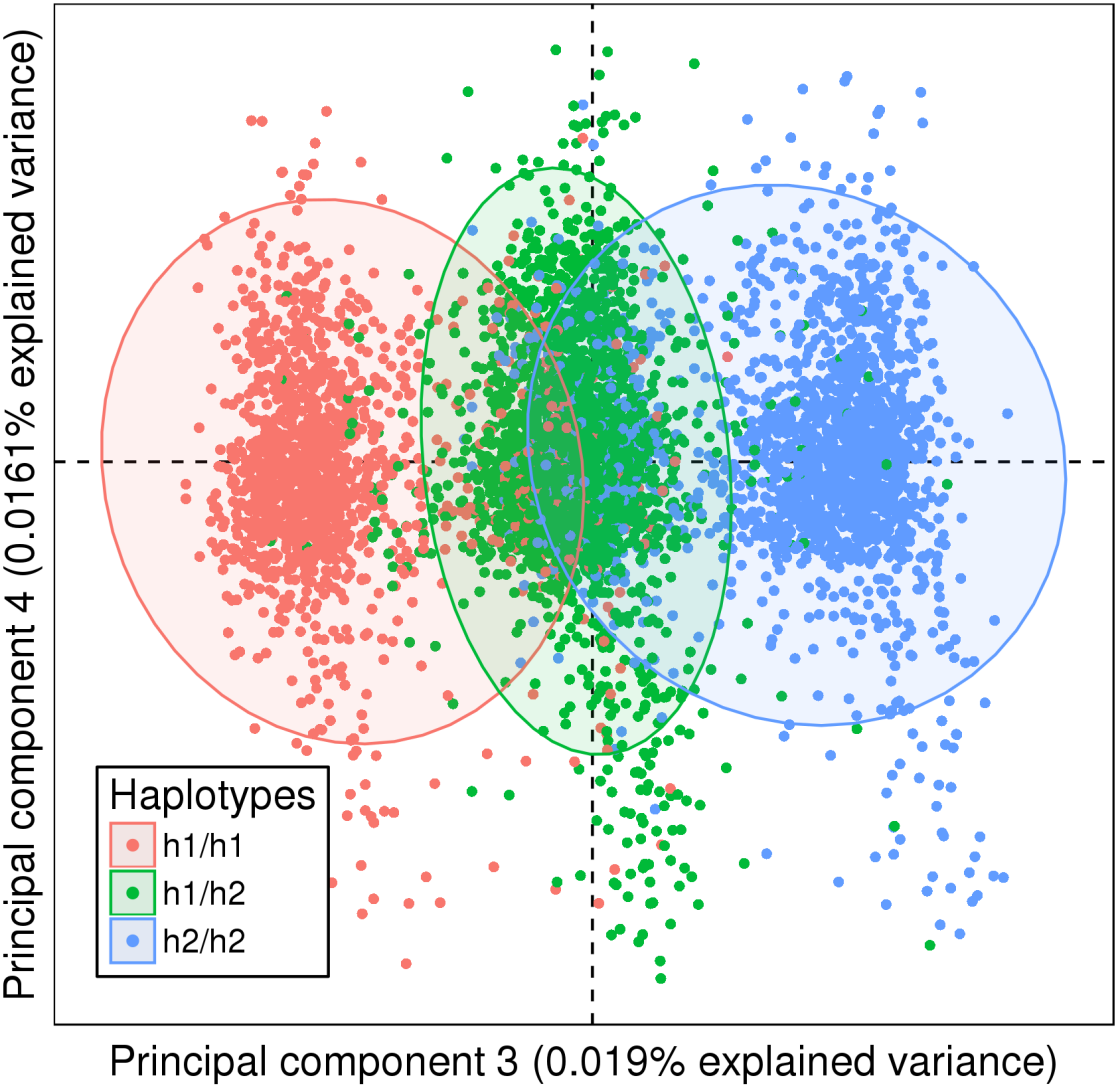
Haplotype summarization of the *8p23* locus. The haplotypes estimated from the 875 selected SNPs from chromosome 8 were best fitted by two groups. The resulting three groups, plotted with 95% confidence ellipses, accurately represented the three clusters in principal component 3 and showed that haplotypes preserved information carried by SNPs.

Then, the 10,422 selected SNPs were reduced to 261 SNPs and 331 haplotypes by the haplotype estimation process described in the methods. For example, on principal component 3, the 875 SNPs from chromosome 8 were summarized by 2 distinct haplotypes h1 and h2. Each subject was assigned a h1/h1, h1/h2, or h2/h2 haplotype combination for this region (Fig 3).

### Ancestry signal reduction

**Fig 4:**
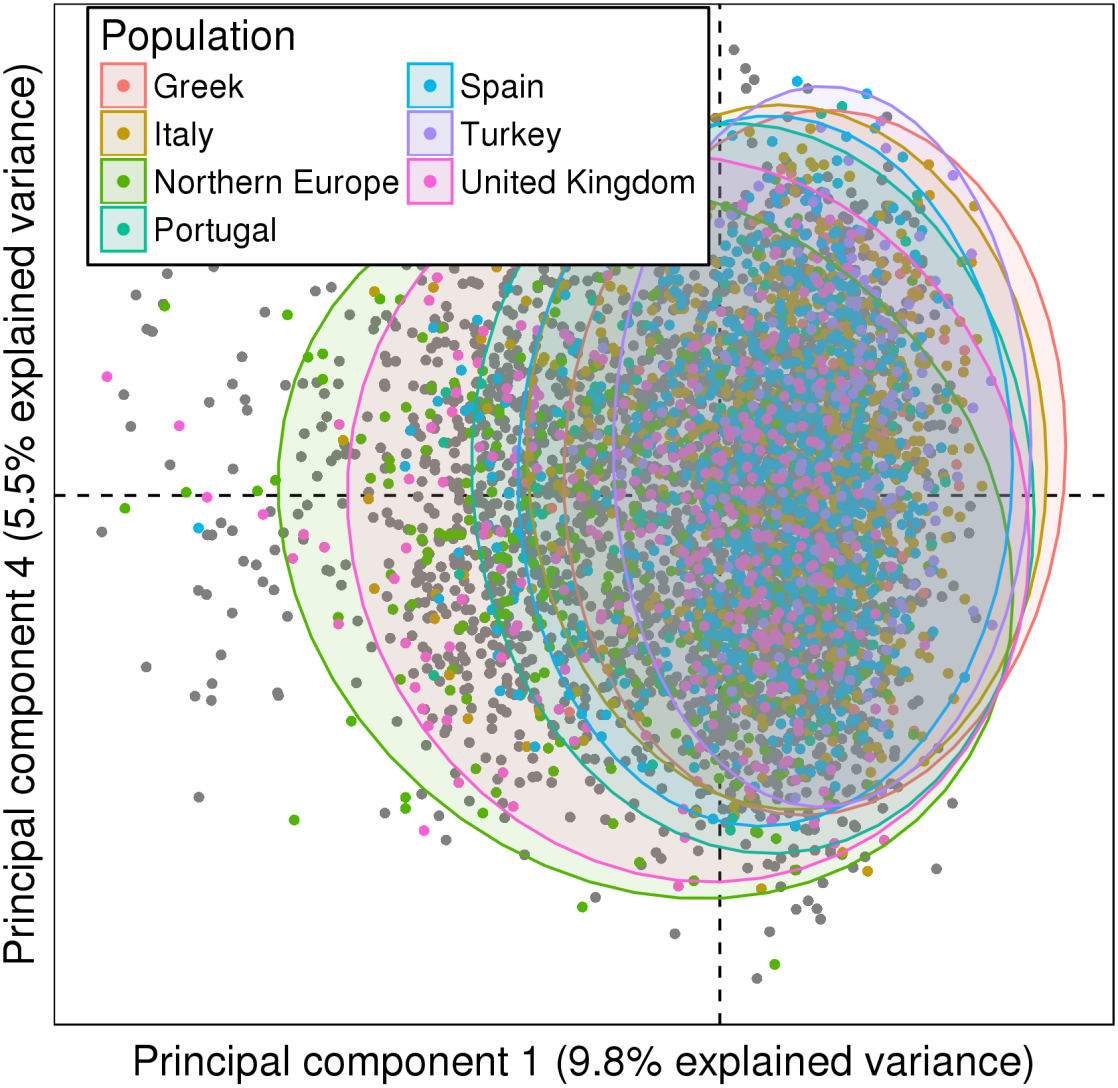
PCA after SNPClust application. First principal components of the PCA on the 261 SNPs and 331 haplotypes from 5,433 patients, with 95% confidence ellipses. The principal components did not discriminate SLE patients from controls.

We applied PCA on the selected SNPs and haplotypes. The first principal components did not cluster patients neither by phenotype nor by centers (Fig 4).

The method therefore reduced the ancestry signal and produced a set of features that could be further investigated for disease relevant signals.

### Benchmark

GWAS have found several loci associated with SADs. However such approaches are impaired by population substructures that can generate false-positives or domestic association signals [4]. In order to evaluate the information conserved by SNPClust and compare it to existing population stratification correction methods, a GWAS was performed on the input dataset, without or with subpopulation structure correction (see methods), and compared with the results of a GWAS performed on the SNPClust selected dataset.

Compared with the GWAS performed directly on the input dataset, the GWAS performed on the SNPClust-processed dataset selected much less SNPs with a nominal p-value < 0.05: 97,066 *vs.* 222. In addition, the initial selection of SNPs by SNPClust reduced the impact of the multiple testing correction: 59% of SNPs on the input dataset with p < 0.05 had a False Discovery Rate (FDR) [12] > 5%, compared to only 30% after SNPClust (Table 1).

**Table 1:** Performances of feature selection methods. Associations of the input dataset without or with population stratification correction by genomic control and Eigenstrat (with 5 and 10 principal components considered) compared with SNPClust.

Feature selection method	Number of p-values < 5%	Number of FDR q-values < 5%	Number of FDR q-values in *HLA* < 5%
(none)	97,066	39,555	951
Genomic control	18,267	271	232
Eigenstrat 5	22,605	117	64
Eigenstrat 10	15,269	28	9
SNPClust	222	156	38

Compared with GWAS with genomic control [9], SNPClust produced 42% less significant associations after multiple testing correction, but 3 times more outside the *HLA* region. Therefore SNPClust has less statistical power than genomic control but is much more effective in finding associations from several loci. Eigenstrat [10] was performed with 5 and 10 principal components. In both cases, SNPClust had more statistical power, with less hits in *HLA* (24% *vs.* 55% and 32%).

Increasing statistical power can increase false positive rate. However SLE has still a large unexplained heritability and increasing the power is a possible step to reduce it by discovering novel markers. Additionally, the FDR multiple testing correction method can also be more stringent to reduce the power and the false positive rate.

The *HLA* locus exhibited a pitfall of GWAS with multiple testing correction: even after TagSNP selection, most associated SNPs were from few loci, due to haplotypes. This had the effect of excluding other loci when multiple testing correction was applied. SNPClust overcame this by summarizing the SNPs from one locus in one haplotype. Associated genes found by SNPClust and Eigenstrat with 5 components were compared. SNPClust had 67 unique genes associated, Eigenstrat had 37, and with an intersection of 1. The associated genes were also compared to previously known genes associated with SADs. The intersection had 1 known gene (*NOTCH4*). Excluding this one, SNPClust had 4 known genes (*MICA*, *MSH*, *PSORS1C1*, *RAD51B*) and Eigenstrat 4 (*ATG5*, *BANK1*, *STAT4*, *TNXB*).

## Discussion

The ancestry information is contained in many SNPs across the genome, and may therefore be present in clinically relevant SNPs, in particular in auto-immune diseases where the *HLA* locus is involved. Therefore removing simply the main known ancestry-informative markers may lead to the removal of clinically relevant SNPs while preserving many SNPs carrying small bits of ancestry information. Approaches considering the first principal components to adjust associations [10] can also result in loss of clinically relevant information because not all the first principal components are associated with ancestry.

SNPClust overcomes these limitations in two steps. First the major contributors to a large number of the first principal components are considered, therefore selecting the markers explaining most of the variance in the dataset. This has the property of preserving the largest sources of genetic variation. Then, the SNPs that could be considered as haplotypes due to their correlation and spatial proximity are summarized. This reduces the relative importance of ancestry information present in many SNPs while preserving the information conveyed by the haplotypes. At the same time, it also reduces the multiple-testing problem.

In the dataset of 379,190 SNPs from SLE patients and controls, the first principal components were associated with ancestry. After application of SNPClust 592 markers remained and the first principal components were not associated to the centers. Therefore the ancestry information and the number of markers were strongly reduced.

This method is also interesting for GWAS, due to the enrichment of ancestry-independent markers tested and the reduced multiple testing problem. When compared to genomic control, 42% less SNPs were associated with the diseases but 10 times more outside the *HLA* locus. Compared to Eigenstrat, SNPClust had more statistical power and a higher ratio of associations outside *HLA*. Therefore SNPClust is useful to find several associated loci without being overwhelmed by strong signals from one single locus such as *HLA*.

SNPClust will be applied to the SNP array data generated in the PRECISESADS project and will possibly enable the use of ancestry-independent genetic information in the reclassification of SADs. It is available as an R package and demonstrated on the public Human Genome Diversity Project dataset [6] at https://github.com/ThomasChln/snpclust.

## Materials and Methods

### Input dataset

The dataset contained 4,212 European SLE patients and 1,221 European healthy controls. It was previously published [13] and approved by ethics committees. No samples were used and records were de-identified. The files were binary PLINK files [14]. They were converted in the Genomic Data Structure format [15].

### Analysis set

Observations with missing value rates above 3% and SNPs with missing value rates above 1% were excluded. An additive genetic model is then used (AA = 0, AB = 1, BB = 2) and SNPs with MAF below 5% were excluded to remove rare variants, which are more prone to genotyping errors. In addition, in order to decrease the required computation time and memory usage, redundant SNPs were removed by applying TagSNP (*r^2^* > 0.8, window of 500,000 base pairs). The missing values were imputed by random sampling of each SNP. Then each SNP was centered and scaled to unit variance.

A total of 5,433 patients and 379,190 SNPs were selected for analysis. This dataset defines our analysis set.

### SNPClust

#### Selection of strong contributors to principal components

PCA was applied on the analysis set. PCA is a dimensionality reduction method, which projects SNPs by linear combination to maximize the variance on successive axes, *i.e.* principal components, while constraining the axes to be orthogonal. SNPs with large absolute projection values, *i.e.* loadings or contributions, to the 100 first principal components were selected.

For each principal component, a Gaussian mixture model [16] with 2 mixture components was fitted to the absolute values of SNP contributions. Only the 3,000 highest absolute contributions were considered for computational performance. In Gaussian mixtures, SNPs have a probability of being assigned to each Gaussian model, from which can be derived a classification uncertainty. Only the strong contributors have null uncertainty, therefore SNPs with a null classification uncertainty were selected. If SNPs were selected from more than 8 chromosomes, the model was fitted with an additional mixture until the condition was satisfied. If the condition was not satisfied after 4 iterations, *i.e.* with 5 mixtures, no SNPs were selected from that principal component (Algorithm 1).

**Algorithm 1** Selection of strong contributors

1: **Input:** PCA rotation matrix *i.e.* SNPs coefficients to principal components

2: **for** *coefficients* **in** *coefficients*_*PC1*_, …, *coefficients*_*PC100*_ **do**

3:  *coefficients* ← 3,000 highest absolute values of *coefficients*

4:  *selection* ← ∅

5:  **for** *n*_*mixtures*_ **in** 2, 3, 4, 5 **do**

6:   *GMM* ← Gaussian mixture model of *n*_*mixtures*_ components on *coefficients*

7:   *selection* ← *coefficients* with null uncertainty classification in *GMM*

8:   **if** Number of chromosomes in *selection* < = 8 **then**

9:    **exit for loop**

10:   **end if**

11:  **end for**

12:  **store** *selection* **in output**

13: **end for**

14: **Output:** *selection*_*PC*1_, …, *selection*_*PC*100_

#### Haplotype summarization

In a second step, in order to summarize large loci, for each principal component, selected SNPs on a same chromosome and closer than 1,000,000 base pairs away were summarized by haplotypes, pairs of binary values. Haplotypes were inferred with the SHAPEIT software [17] which uses hidden Markov models and has linear complexity with the number of SNPs. Haplotypes were then grouped in 2 classes by Gaussian mixture models. Correlated haplotypes were removed when they were on the same chromosome and the squared correlation was above 0.8 (Algorithm 2).

**Algorithm 2** Summarization of physically close SNPs

1: **Input:** Selected SNPs for the 100 first principal components

2: *haplotypes* ← ∅

3: **for** *SNPs* **in** *selection*_*PC*1_, …, *selection*_*PC*100_ **do**

4:  **for** *locus* **in** groups of SNPs closer than 1,000,000 base pairs in *SNPs* **do**

5:   *haplotype* ← Haplotype estimation of *locus*

6:   *SNPs* ← Exclude *locus* from *SNPs*

7:   **store** *haplotype* **in** *haplotypes*

8:  **end for**

9: **end for**

10: **for** *haplotypes* **in** groups of *haplotypes* from same chromosome **do**

11:  *haplotypes* ← Squared correlation threshold of 0.8 on *haplotypes*

12: **end for**

13: **Output:** Union of *haplotypes* and *SNPs*

### Benchmark

To evaluate the SNPClust algorithm, we performed a GWAS on the SNPClust selected dataset and on the input dataset with and without population stratification correction. First, a generalized linear model with a binomial error distribution was fitted to each SNP and haplotype to predict the disease of patients. Type 2 Analysis of Variance was then applied to obtain p-values.

Multiple testing correction was performed with FDR, and results were compared with the outcome of our method. Then, two population stratification correction methods were tested, genomic control and Eigenstrat.
